# Supplementary material for: Expression of ERBB Family Members as Predictive Markers of Prostate Cancer Progression and Mortality
Source: Cancers (Basel). 2021 Apr 2;13(7):1688. doi: 10.3390/cancers13071688 (PMC8038288; doi:10.3390/cancers13071688)
Supplement: Supplementary file 1 [file cancers-13-01688-s001.pdf]

cancers MDPI

Supplementary Materials:

Expression of ERBB family members as predictive markers of prostate cancer progression and mortality

Sylvie Clairefond, Véronique Ouellet, Benjamin Péant, Véronique Barrès, Pierre I. Karakiewicz, Anne-Marie Mes-Masson and Fred Saad

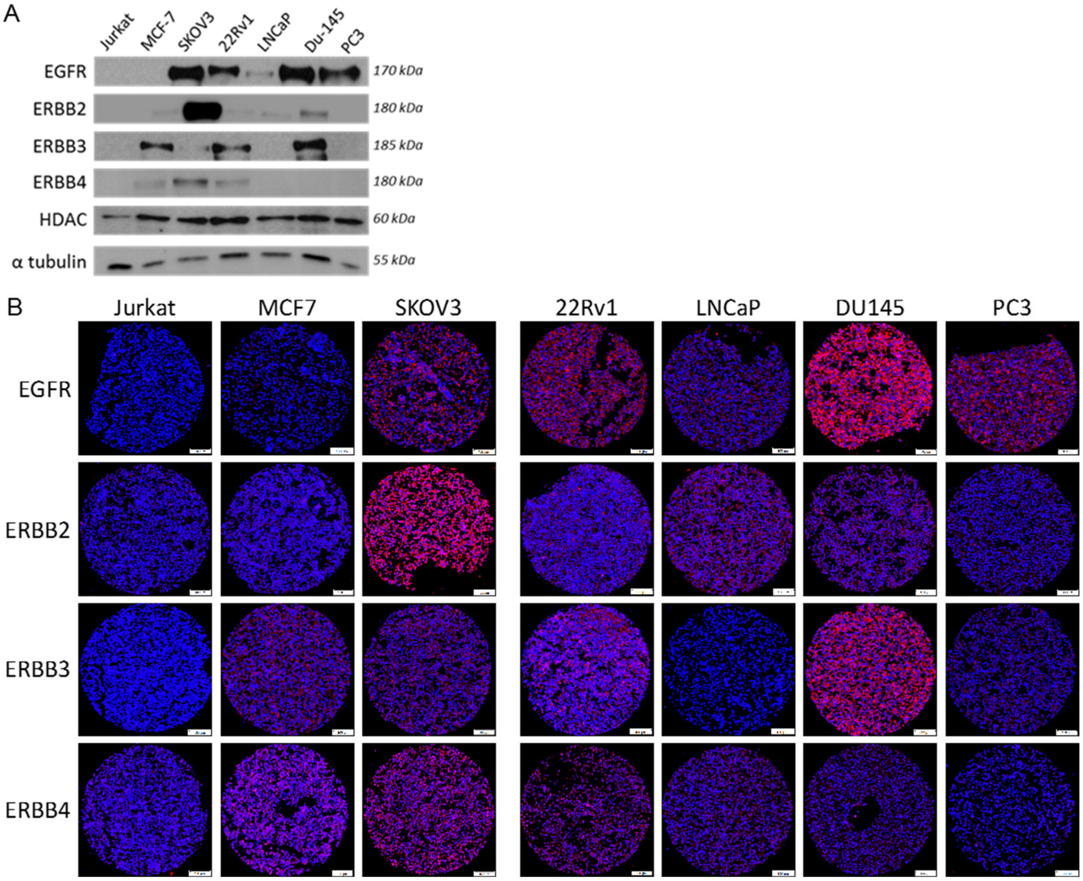

Figure S1. Evaluation of antibody specificity for all ERBB family members. (A) Expression of EGFR, ERRB2, ERBB3, and ERBB4 evaluated by western blot in different cell lines. α-tubulin or HDAC served as a loading control for Western blots. (B) Representative staining for each protein in different paraffin-embedded cell lines. DAPI (blue) and protein of interest (red). The scale bar at 100 μm is for the whole image.

A

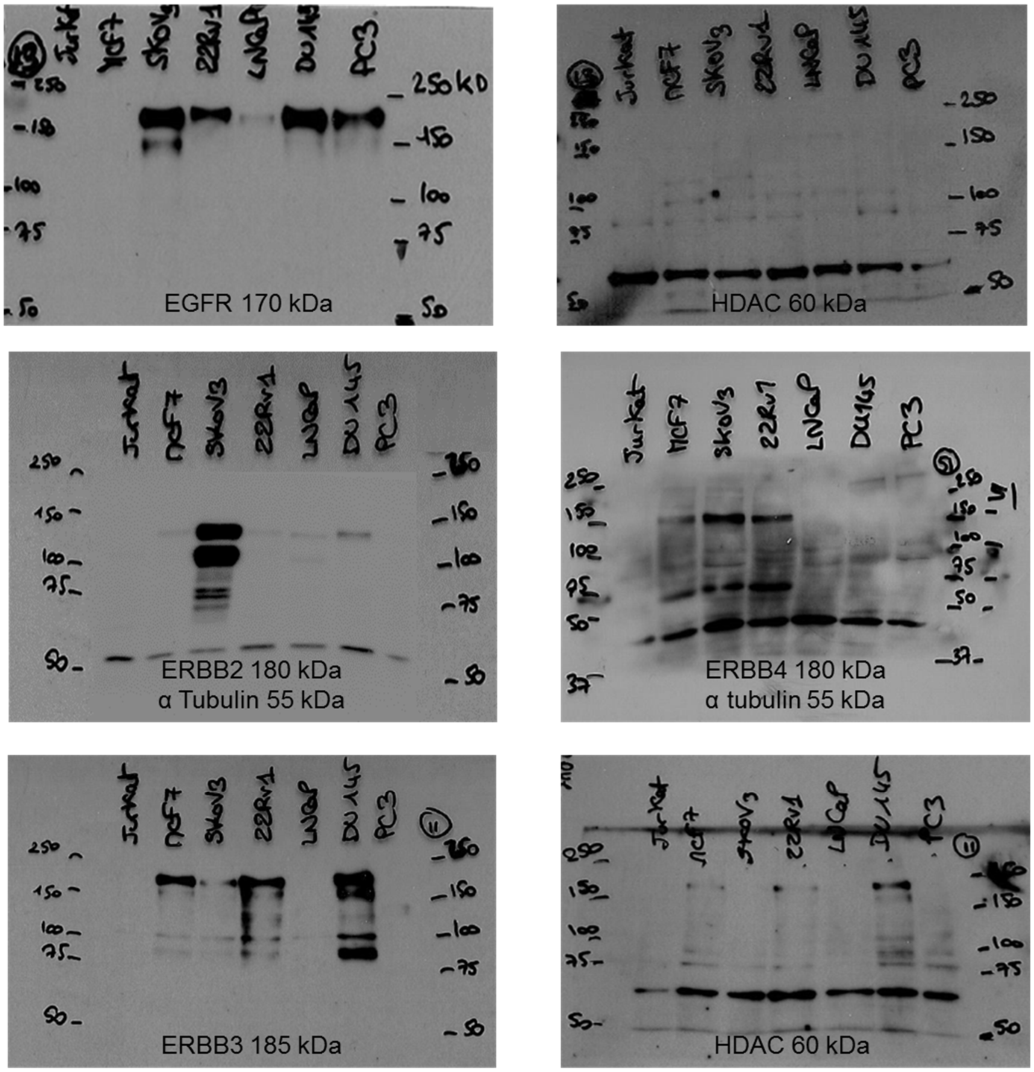

B

	Jurkat	MCF-7	SKOV3	22Rv1	LNCaP	DU145	PC3
EGFR	0.34	0.27	1.63	0.83	0.61	1.79	1.40
ERBB2	0.41	0.95	1.67	0.78	0.62	0.88	0.15
ERBB3	0.32	0.94	0.83	1.24	0.54	1.50	0.36
ERBB4	0.27	0.72	0.82	0.80	0.35	0.43	0.38

Figure S2. Whole Western blots of ERBB expression in PC cell lines. (A) Detection of EGFR, ERBB2, ERBB3 and ERBB4 in whole cell lysates in PC cell lines, with α-Tubulin or HDAC as a control. (B) Intensity ratio of biomarkers for each cell line, normalized with α-Tubulin or HDAC.
